# A novel strategy to avoid sensitivity loss in pooled testing for SARS-CoV-2 surveillance: validation using nasopharyngeal swab and saliva samples

**DOI:** 10.3389/fpubh.2023.1190308

**Published:** 2023-08-10

**Authors:** Georgia G. Millward, Shane M. Popelka, Anthony G. Gutierrez, William J. Kowallis, Robert L. von Tersch, Subrahmanyam V. Yerramilli

**Affiliations:** Emerging Biological Threats Branch, Molecular Biology Division, Laboratory Sciences, Defense Centers for Public Health - Aberdeen “Formerly the Army Public Health Center”, Aberdeen Proving Ground, Edgewood, MD, United States

**Keywords:** pooled testing, SARS-CoV-2, real-time PCR, surveillance screening, asymptomatic carriers

## Abstract

At the peak of the COVID-19 pandemic, pooled surveillance strategies were employed to alleviate the overwhelming demand for clinical testing facilities. A major drawback of most pooled-testing methods is the dilution of positive samples, which leads to a loss of detection sensitivity and the potential for false negatives. We developed a novel pooling strategy that compensates for the initial dilution with an appropriate concentration during nucleic acid extraction and real-time PCR. We demonstrated the proof of principle using laboratory-created 10-sample pools with one positive and corresponding individual positive samples by spiking a known amount of heat-inactivated SARS-CoV-2 into viral transport medium (VTM) or pooled negative saliva. No Ct difference was observed between a 10-sample pool with one positive vs. the corresponding individually analyzed positive sample by this method, suggesting that there is no detectable loss of sensitivity. We further validated this approach by using nasopharyngeal swab (NPS) specimens and showed that there is no loss of sensitivity. Serial dilutions of the virus were spiked into VTM and pooled with negative saliva in simulated 10-sample pools containing one positive to determine the LOD and process efficiency of this pooling methodology. The LOD of this approach was 10 copies/PCR, and the process efficiencies are ~95%−103% for N1 and ~87%−98% for N2 with samples in different matrices and with two different master mixes tested. Relative to TaqPath 1-step master mix, the TaqMan Fast Virus 1-Step master mix showed better sensitivity for the N2 assay, while the N1 assay showed no Ct difference. Our pooled testing strategy can facilitate large-scale, cost-effective SARS-CoV-2 surveillance screening and maintain the same level of sensitivity when analyzed individually or in a pool. This approach is highly relevant for public health surveillance efforts aimed at mitigating SARS-CoV-2 spread.

## Introduction

The coronavirus disease 2019 (COVID-19) outbreak caused by severe acute respiratory syndrome coronavirus 2 (SARS-CoV-2) first appeared in Wuhan, China, in 2019 and spread rapidly into a global pandemic (1–4). This outbreak seriously impacted public health worldwide and caused an unprecedented demand for SARS-CoV-2 diagnostic testing resources. The most widely used diagnostic test to detect the presence of viral RNA is the highly sensitive quantitative real-time polymerase chain reaction (qPCR), the gold standard for SARS-CoV-2 detection. Very early on in the pandemic, a test for this virus developed by the US Centers for Disease Control and Prevention (CDC) (5, 6) was approved by the Food and Drug Administration (FDA) for emergency use (7) by laboratories.

While the focus has been on SARS-CoV-2 patients with symptoms (symptomatic), infected individuals who do not show any symptoms (pre-symptomatic and asymptomatic carriers) have been a substantial source of all reported infections (8–10). Transmission without symptoms has been a major contributor to the rapid spread of the virus (11–14). This highlights the importance of public health efforts to conduct widespread surveillance testing of asymptomatic individuals, particularly in close-quarter communities such as military installations, schools, health-care facilities, and nursing homes. Large-scale testing presents a major challenge due to the number of individuals (community size) to be tested on a regular basis to mitigate the risk. This imposes a serious burden on much-needed resources and on test centers that are already at their maximum capacity with symptomatic and other medical testing. Sample pooling-based group testing approaches can expand analytical capacity, allowing large-scale surveillance screening of asymptomatic carriers and enhancing public health intervention efforts.

Adaptive group testing, first described by Dorfman (15), is a two-stage hierarchical testing strategy in which samples are tested first in multi-sample pools, and subsequently, individual samples from positive pools are analyzed to identify the positives. Pools that tested negative indicate that all the samples in that pool are negative and that no further testing is necessary. Sample pooling reduces the total number of tests and increases the overall throughput. This has been the most widely used approach, and several laboratories have employed this strategy for real-time PCR detection of SARS-CoV-2. The most preferred pooling strategy has been to mix individual samples prior to RNA extraction and real-time PCR (16–21), while other methods, such as swab-level pooling at the time of collection (22–24) and RNA-level pooling (25, 26), were also used to increase the analytical capacity and save resources. Optimal pool size and the effectiveness of pooling are dependent on the disease prevalence in a population and assay sensitivity (16, 17, 27–29). The lower the prevalence, the higher the benefits of pooling; however, prevalence is not static and could change very quickly. A built-in flexibility to adjust the pool size is very important for any pooling methodology to accommodate changes in prevalence while maintaining sensitivity.

NPS specimens have been the gold standard for SARS-CoV-2 diagnostic detection and group testing approaches. Collecting NPS specimens is costly, time-consuming, and requires trained personnel with personal protective equipment (PPE). Less expensive and easy to collect alternate specimen types, such as saliva (30, 31) and saline-gargle (32–34), are emerging as valid choices for real-time PCR-based SARS-CoV-2 detection and can simplify surveillance efforts. The collection of these specimens is non-invasive, can easily be self-collected, and does not require trained personnel or PPE. The use of saliva as a sample for detecting the virus is a major turning point in surveillance testing (35) and is a compelling alternative to NPS (36). SARS-CoV-2 RNA in saliva is relatively stable (37) and saliva has comparable sensitivity to NPS in molecular detection of the virus (30, 38–41). Several groups have effectively used saliva in pooled surveillance testing of SARS-CoV-2 (42–48).

Although group testing enhances analytical capacity, a major drawback of most pooled testing approaches is loss of sensitivity due to sample dilution (16–19, 25, 26, 40, 43, 45, 47). A 10-fold dilution can increase the Ct value by ~3.33 (49). Consequently, specimens with viral loads close to the LOD are likely to become false negatives upon sample pooling due to this dilution effect. Here we present proof-of-concept data in support of a pooled testing strategy for up to 10 samples without any loss of sensitivity due to dilution (and avoiding the Ct increase). This method is bench-marked with the CDC protocol for single-sample analysis (5, 6). This approach compensates for the initial 10-fold dilution with an appropriate seamless concentration during subsequent nucleic acid extraction and real-time PCR steps. We validated this method with NPS as well as with self-collected saliva and determined the process efficiency and LOD. This pooled testing strategy can be utilized very effectively by any laboratory engaged in large-scale SARS-CoV-2 surveillance screening. This pooling strategy is extremely beneficial and effective in providing much-needed support to epidemiological monitoring during devastating pandemic situations such as COVID-19.

## Materials and methods

### Reagents

MagMAX™ Viral/Pathogen Nucleic Acid Isolation Kit (MVP II), Phosphate Buffered Saline (PBS; GIBCO, ThermoFisher Scientific, Cat # 10010031), TaqPath™ 1-Step RT-qPCR Master Mix, CG (4×), and TaqMan™ FAST Virus 1-step Master Mix (4×) were purchased from ThermoFisher Scientific. Heat-Inactivated SARS-Related Coronavirus 2, Isolate USA-WA1/2020 (Cat# NR-52286), and Quantitative PCR (qPCR) control RNA from Heat-Inactivated SARS-Related Coronavirus 2, Isolate USA-WA1/2020 (Cat# NR-52347) provided by the BEI Resources Repository (www.beiresources.org) were used in our experiments as the reference viral preparation and positive qPCR control, respectively. The viral transport medium (VTM) was prepared as described in Centers for Disease Control and Prevention SOP#: DSR-052-05 (50). Proteinase K solution (Cat# 1019499) was purchased from Qiagen, Inc.

### MS2 bacteriophage

MS2 bacteriophage (5 × 10^10^ viral particles (copies)/ml; Cat# 0810274) was purchased from ZeptoMetrix, Inc. An aliquot of this stock was diluted to a working stock of 5 × 10^6^ copies/ml in PBS. Each individual sample (when the samples were analyzed individually) or a 10-sample pool (when the samples were analyzed as pools) was spiked with 2 μl (~10,000 copies) of this working stock at the time of nucleic acid extraction. This serves as extraction control. MS2 Phage RNA from Roche (Cat # 10165948001, 10 A260 Units) was used as positive control RNA for real-time PCR (~1–2 pg in a 20-μl real-time PCR reaction).

### Primers/probes

2019-nCoV RUO Kit (Cat# 10006713), consisting of 2019-nCoV_N1, 2019-nCoV_N2, and Hs_RP (RNAse P), was purchased from Integrated DNA Technologies (IDT). MS2 Bacteriophage RNA replicase β-chain gene-specific RT-qPCR Primers and Probe sequences published (51) were used. MS2-RB-FWD primer: 5′-GCT CTG AGA GCG GCT CTA TTG-3′, MS2-RB-REV primer: 5′-CGT TAT AGC GGA CCG CGT-3′, and MS2-RB probe: 5′-/56-FAM/CCG AGA CCA/ZEN/ATG TGC GCC GTG/3IABkFQ/-3′ also from IDT. For the MS2RB assay, the final concentration for FWD and REV primers was 400 nM, and the probe was used at 250 nM in real-time PCR (20-μl final volume). We prepared a 10 × concentrated primer/probe mix and used a 2 μl/20 μl reaction.

### Sample collection

NPS specimens were collected into VTM from asymptomatic service members by trained personnel with personal protective equipment (PPE) and following appropriate safety procedures. The samples were shipped on ice to the Department of the Army (DA) Public Health Center (APHC), which has since been renamed the Defense Centers for Public Health-Aberdeen (DCPH-A). The samples were shipped on ice and used for pooling and subsequent testing. Positive pools, each having 10 pool-specific individual de-identified samples, were kindly provided by Dr. Robyn Nadolny (Vector-Borne Diseases Branch, DCPH-A).

### SARS-CoV-2 negative saliva

Self-collected, de-identified saliva samples (a batch of 10 individuals at any given time and 4–5 ml saliva from each) were obtained from healthy volunteers (internal staff) with their consent and permission from the Office of Human Protection (OHP) following biosafety guidelines. We used sterile, unmarked 30-ml freestanding screw-capped tubes (Evergreen Labware products, Cat# 222-3530-G80) for saliva collection.

### Heat inactivation

All the unopened specimens (NPS and saliva) were heat inactivated to render the SARS-CoV-2 (if any present in the specimen) non-infectious by placing the tubes in a 65°C bath for 30 min. These tubes were moved to a biosafety cabinet (BSC, class II) for added safety and processed further.

In the case of heat-inactivated saliva specimens, an aliquot (100 μl) of each was treated with Proteinase K solution (10 μl) at room temperature for 15 min with occasional gentle mixing to reduce the viscosity. Proteinase K-treated saliva (100 μl) was combined with 300 μl of PBS, spiked with 5 μl of MS2-EC, and processed for nucleic acid extraction. The elution volume was 80 μl. Extracted RNA (10 μl) was used in real-time PCR to test for SARS-CoV-2-specific N1 and N2. Saliva specimens negative for SARS-CoV-2 were used to prepare pooled negative saliva and stored at −80°C in aliquots until use. This pooled saliva was used for subsequent validation studies by spiking known copies of heat-inactivated SARS-CoV-2.

### Pooling method validation using simulated pools

Heat-inactivated SARS-CoV-2 virus stock was prepared (3 × 10^4^ copies/ml) in specimen matrix (VTM or pooled negative saliva), and a 400 μl (12,000 copies) aliquot was used as the starting material for un-pooled individual samples, while only 40 μl (1,200 copies) combined with 360 μl of specimen matrix was used for the 10-sample pool (relative to un-pooled, it is 1:10 diluted). For each condition, five replicates were prepared and processed for nucleic acid extraction. At the elution step, the un-pooled sample was eluted into 400 μl (maintaining a 1:1 relation between the input and the elution volumes), while for the pools, it was 80 μl (leading to a 5× concentration). At the real-time PCR step, 5 μl of the extracted nucleic acid was used from the un-pooled sample while 10 μl (2× volume) was used from the simulated pool, compensating for the initial 10× dilution by appropriately concentrating at the time of nucleic acid extraction and at the real-time PCR steps. As a result, in both cases, there were approximately 150 copies per qPCR reaction (no dilution effect).

### Validation using surveillance samples from asymptomatic individuals

We further validated this pooling approach using de-identified NPS surveillance samples. Sample pools (of 10) were prepared using individual heat-inactivated NPS specimens (collected in VTM) by aliquoting 100 μl of each specimen using a P200 pipettor with an extra-long filtered pipette tip (cat# 2160P-05-HR, ART-Reach barrier tip, sterile, ThermoFisher Scientific) into a 1.5 ml Eppendorf tube to make a 10-sample pool. The final volume at this stage was 1 ml. When the number of samples for pooling is <10, an appropriate volume of VTM is added to make up the final volume of 1 ml. Using a 1-ml pipettor with a filtered tip, the pooled sample was gently mixed to make it homogeneous. All these steps were performed in a Biosafety Cabinet (BSC, Class II), taking appropriate precautions. A 400 μl aliquot from each pool was spiked with MS2 bacteriophage as an extraction control (MS2-EC) and prepared for nucleic acid extraction. The remaining pooled sample (600 μl) and the corresponding un-pooled individual specimens were stored at −80°C. Nucleic acids were eluted (in 80 μl), and 10 μl/target was used in real-time PCR. Individual NPS specimens that tested negative were combined to prepare pooled Negative NPS and stored at −80°C until use.

### Individual samples from positive pools were analyzed using two approaches

(a) A volume of 400 μl of the individual sample spiked with MS2-EC was used for nucleic acid extraction. In this case, the elution volume was 400 μl and 5 μl was used for real-time PCR. In doing so, the MS2-EC Ct values would be higher in individual samples than the corresponding MS2-Ct values in the 10-sample pool. (b) A volume of 40 μl of the individual sample was mixed with 360 μl of VTM and spiked with MS2. Following the nucleic acid extraction, the elution volume was 80 and 10 μl was used for real-time PCR. In this case, the MS2-Ct values were very similar between the pool and the individual samples. This latter approach uses less specimen (volume) and minimizes the wastage of potentially valuable specimens.

### Nucleic acid extraction

Nucleic acids were extracted using the MagMAX™ Viral/Pathogen Nucleic Acid Kit (MVP II) on a MagMAX 96 instrument. The starting volume was 400 μl, and samples <400 μl were adjusted to 400 μl by adding the appropriate volume of VTM or pooled negative saliva, depending on the experiment. For pooled samples, the starting sample volume was 400 μl (a pool of 10 samples, 40 μl each), and the elution volume was 80 μl. For un-pooled individual samples, the ratio of input specimen volume to elution volume was maintained at a 1:1 ratio. For example, for an input sample volume of 100 μl adjusted to 400 μl with specimen medium, the elution volume used was also 100 μl.

### Real-time PCR

Each real-time PCR reaction (20 μl) consisted of 5 μl of TaqPath 1-step RT-qPCR master mix or TaqMan FAST virus 1-step RT-qPCR master mix (4×), primer probe mix (1.5 μl of 2019-nCoV_N1 or 2019-nCoV_N2 specific mixes from IDT), and 5 μl un-pooled extracted RNA or 10 μl of pooled extracted RNA. The final reaction volume was adjusted to 20 μl with nuclease-free water. For the MS2-EC assay, 2 μl of 10× primer probe pre-mix was used for a 20-μl reaction. Real-time qPCR cycling conditions were as described in the CDC manual (5). Quantitative PCR (qPCR) control RNA (25–30 copies/qPCR) was used as the positive control with an appropriate negative control in all experiments.

## Results

### Validation of the pooling method

#### Pooling strategy

**Figure 1:**
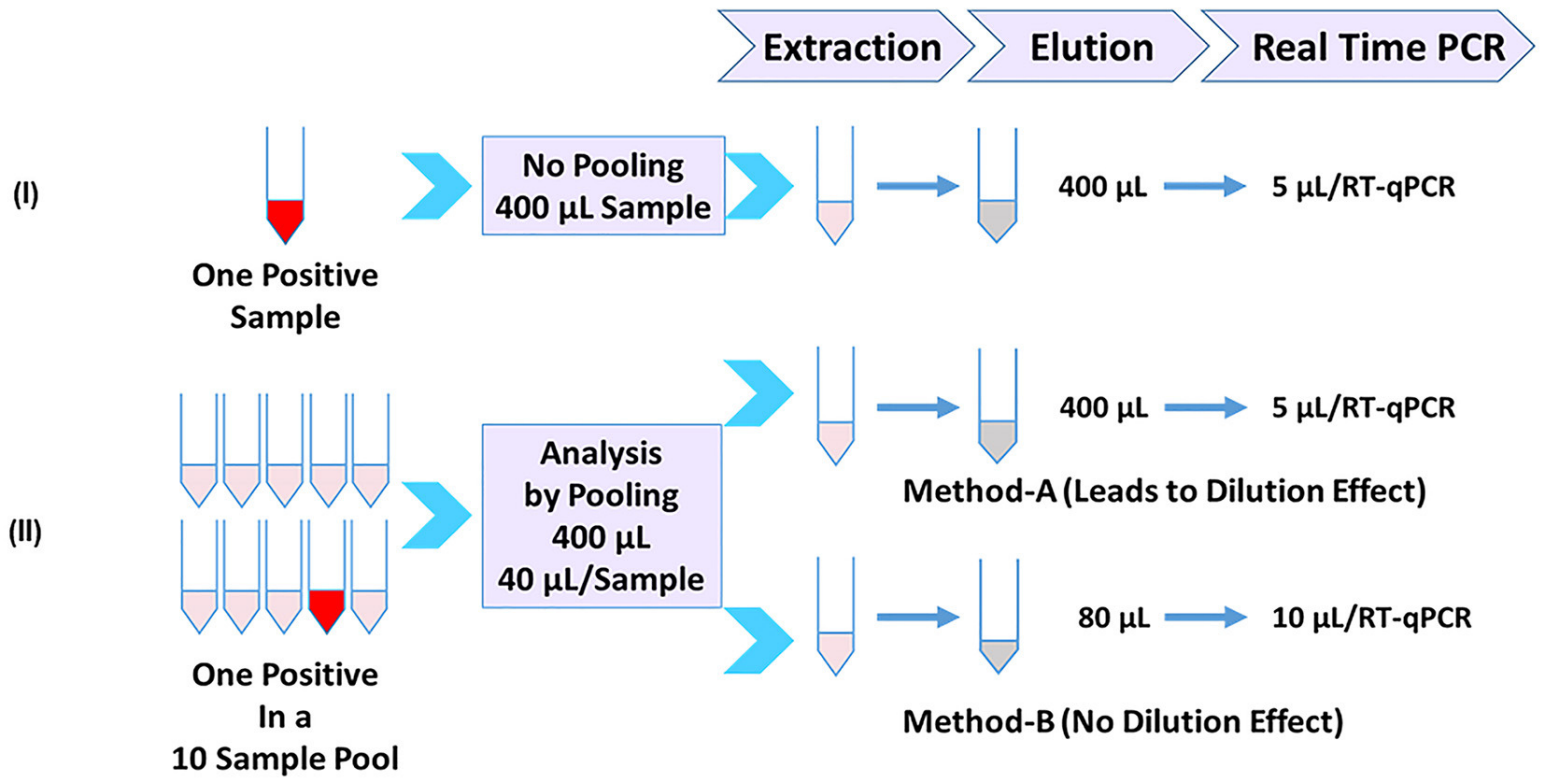
Sample-pooling strategies in relation to individual specimen analysis. **(Panel-I)** Workflow for single sample analysis based on CDC protocol (5) as a benchmark, reflecting a 1:1 relationship between the sample input volume and the elution volume (400 μl in this case). Each target was tested using 5 μl of extracted RNA. **(Panel-II)** Depicts two different approaches for pooling (10 samples) consisting of one positive. Method-A combines 1/10 volume (40 μl) of each sample and processes it as in Panel-I. A positive sample generated by this pooling approach will reflect a dilution effect and loss of sensitivity. Method-B (Present study), the initial dilution is balanced by an appropriate concentration during the nucleic acid elution step and at the real-time RT-PCR step. As a result, the positive sample in the pool doesn't show any dilution effect.

An outline of analyzing samples individually or in pools of 10 for the detection of SARS-CoV-2 is depicted in Figure 1. Panel-I depicts the individual sample analysis workflow, which is based on the CDC 2019-Novel Coronavirus (2019-nCoV) real-time RT-PCR Diagnostic Panel (5) as a benchmark and reflects a 1:1 relationship between the sample input volume and the elution volume (400 μl in this case). SARS-CoV-2-specific targets N1 and N2 are tested using the extracted nucleic acid (5 μl/target) by real-time PCR. Two different 10-sample pooling approaches can be used (Panel-II) by combining 1/10 volume of each sample (40 μl each) to be used to achieve a final input volume of 400 μl for subsequent steps. Nucleic acids are eluted, maintaining the same volume as the input volume (400 μl), and used (5 μl) in real-time PCR as above. Such a pooling approach (Method-A) would lead to dilution and loss of sensitivity. However, the initial 10× sample dilution is balanced (Method-B) by a 5× concentration during the nucleic acid elution step and used a 2× volume of extracted RNA (10 μl) in the subsequent RT-qPCR. As a result, the initial dilution is neutralized, and each sample in the pool performs as if it were a single sample without any dilution effect. It is also possible to directly achieve the 10× concentration at the time of nucleic acid elution depending on the extraction platform used, such as MagNA Pure 96, which can allow the use of an input volume of 1 ml and an elution volume of 0.1 ml, in which case one can use only 5 μl/PCR. We used MagMAX 96 with a starting volume of 400 μl and an elution volume of 80 μl for pools, resulting in a 5× concentration. Using a volume of 10 μl in each qPCR reaction would essentially result in a 10× concentration. This is the basis of this study in this article.

#### Validation by simulated pooling

We validated this pooling strategy through a simulated pooling experiment. We used laboratory-prepared 10-sample pools containing one positive sample and a corresponding individual positive sample by spiking fixed copies of SARS-CoV-2 into specimen medium. These samples were processed accordingly for real-time qPCR analysis, and the resultant Ct values of the pool for N1 and N2 are comparable to the corresponding target-specific Ct values of the un-pooled single sample (Table 1A). If there is a dilution effect, a 10-sample pool would have resulted in a Ct increase of ~3.33 relative to an individually analyzed sample; however, we did not see such an increase in Ct upon pooling. These results suggest that a single positive sample in a background of nine negative samples analyzed individually yielded very comparable results, showing that this approach avoided sensitivity loss due to pooling. We also tested this approach with two different master mixes, and the results of N1 and N2 for a given master mix are comparable to the corresponding target-specific Ct values in a simulated pool vs. a single un-pooled sample (Table 1A), indicating that this approach has not resulted in a Ct increase (~3.33) typical for the dilution effect (10-sample pooling). Our results also show that this pooling approach can be used not only with NP swabs but also with saliva (Table 1B) specimens.

**Table 1A:** Simulated pooling experiment.

	**VTM**			
	**TaqPath 1-step Master Mix**.		**TaqMan FAST Virus 1-step Master Mix**.	
	**Pool**	**Individual**	**Pool**	**Individual**
	**Ct mean** ± **SD**	**Ct mean** ± **SD**	**Ct mean** ± **SD**	**Ct mean** ± **SD**
**N1**	30.58 ± 0.27	31.38 ± 0.33	30.21 ± 0.15	30.60 ± 0.19
**N2**	33.57 ± 0.37	33.38 ± 0.30	30.44 ± 0.10	30.78 ± 0.36

**Table 1B:** 

	**VTM**		**Saliva**	
	**TaqMan FAST Virus 1-step Master Mix**.			
	**Pool**	**Individual**	**Pool**	**Individual**
	**Ct mean** ± **SD**	**Ct mean** ± **SD**	**Ct mean** ± **SD**	**Ct mean** ± **SD**
**N1**	29.94 ± 0.27	29.67 ± 0.22	29.95 ± 0.22	29.31 ± 0.16
**N2**	29.71 ± 0.15	29.69 ± 0.17	30.11 ± 0.18	29.23 ± 0.11

Simulated pools and corresponding individual samples were prepared using heat-inactivated SARS-CoV-2, and the nucleic acids were analyzed by real-time PCR. The data presented here are the mean Ct and ± SD of five replicate samples (150 copies/qPCR) from pooled and individual samples prepared in VTM (Table A) and in the saliva matrix (Table B). Real-time PCR was performed on QuantStudio-6 (Table A) and ABI 7500 Dx (Table B).

#### Validation using surveillance samples

Asymptomatic surveillance samples (NPS) analyzed individually or as 10-sample pools also showed no loss of sensitivity due to pooling. The Ct values obtained with the positive 10-sample pools are highly comparable to the corresponding individually tested positive sample(s), further validating that there is no dilution effect (Table 2). This agrees with what we observed using a laboratory-prepared simulated pool vs. an individual sample scenario. Some pools resulted in multiple positive samples (Table 3), and in such situations, the Ct value of the pool reflected the individual sample with the lowest Ct in that pool. MS2-EC Ct values of individually analyzed samples remain comparable to those of the pool or higher. Depending on the approach used for individual sample analysis (methods), MS2 Ct values are 2–3 Ct higher for some individual samples relative to the corresponding pool (P9, P15, P17, and P18 of Table 2; P30, P32, and P1 of Table 3). Based on our studies, we recommend the approach that uses only 40 μl of an individual sample, potentially saving the specimen for other studies.

**Table 2:** Surveillance samples pooled vs. individuals with one positive in a 10-sample pool.

**Pool ID**	**Positive individual ID**	**Ct-N1**		**Ct-N2**		**Ct-MS2**	
		**Pool**	**Individual**	**Pool**	**Individual**	**Pool**	**Individual**
P9	P9-1	31.26	30.89	31.59	31.24	28.74	30.15
P15	P15-1	28.26	28.16	28.91	28.31	28.98	31.87
P17	P17-8	29.95	29.38	30.50	29.83	28.34	30.87
P18	P18-6	33.98	34.63	33.29	34.40	28.63	31.42
P2	P2-2	33.48	33.93	33.80	33.83	28.15	27.23
P6	P6-2	34.38	35.13	34.81	35.02	27.82	27.60
P10	P10-7	33.45	32.84	34.61	34.34	27.65	27.28
P12	P12-9	32.30	32.83	32.69	33.49	27.66	27.82
P13	P13-4	35.85	35.91	36.23	35.70	27.63	27.62
P14	P14-1	33.27	32.97	33.51	33.20	—	27.78
P19	P19-10	31.49	31.70	31.72	31.81	28.10	27.71
P25	P25-3	25.76	26.11	26.19	26.34	27.68	27.90
P29	P29-8	35.49	35.55	34.46	34.86	27.44	28.00
P27	P27-3	35.48	35.98	35.77	37.01	27.95	27.86

A panel of deidentified surveillance samples (NPS in VTM) consisting of positive pools and corresponding individuals was used. This table shows target-specific Ct values for each pool and its corresponding positive individual sample.

MS2-EC Ct values for pools P9, P15, P17, and P18 are lower by ~2–3 Ct in comparison to the MS2-EC Ct of the corresponding individual sample based on the approach used in individual sample analysis.

–, no signal.

**Table 3:** Surveillance samples pooled vs. individuals with more than one positive in a 10-sample pool.

**Pool-ID**	**Positives in the pool**	**Analysis**	**Individual ID**	**Ct-N1**	**Ct-N2**	**Ct-MS2**
P7	2	Pool	**27.40**	**27.65**	27.88
		Individual	P7-3	**27.60**	**27.94**	27.92
		Individual	P7-4	29.45	29.82	27.91
P8	2	Pool	30.97	31.66	28.25
		Individual	P8-4	31.41	31.59	28.83
		Individual	P8-9	**30.83**	**31.61**	30.55
P21	2	Pool	**29.71**	**29.89**	28.33
		Individual	P21-8	32.25	32.15	27.58
		Individual	P21-10	**30.40**	**30.72**	27.89
P16	2	Pool	**23.28**	**23.62**	27.57
		Individual	P16-1	36.29	38.12	27.67
		Individual	P16-7	—	37.34	27.65
		Individual	P16-9	**23.36**	**23.62**	27.78
P30	2	Pool	**31.84**	**32.37**	28.38
		Individual	P30-4	**32.54**	**32.32**	30.46
		Individual	P30-7	37.66	37.03	30.35
P32	2	Pool	**16.48**	**16.46**	28.29
		Individual	P19-5	**16.62**	**16.66**	31.48
		Individual	P19-7	37.47	—	31.30
		Individual	P19-8	34.01	35.18	31.26
P1	3	Pool	**25.76**	**26.03**	27.77
		Individual	P1-2	28.97	29.71	30.08
		Individual	P1-3	35.89	35.10	29.70
		Individual	P1-6	37.60	—	29.87
		Individual	P1-8	**25.54**	**26.07**	30.61
P31	5	Pool	**22.97**	**23.29**	27.52
		Individual	P31-3	**23.18**	**23.49**	27.40
		Individual	P31-5	28.25	28.27	27.72
		Individual	P31-7	32.72	32.62	27.60
		Individual	P31-8	32.25	31.67	27.55
		Individual	P31-10	29.55	30.07	27.83

Ct values for pools and corresponding positive individual samples where a given pool has two or more positive samples. In all these cases, the N1 and N2 Ct values of the pool (in bold) represented the corresponding N1 and N2 Ct values of the individual sample with the lowest Ct (also in bold) in that pool.

MS2-EC Ct values for pools P30, P32, and P1 are lower by ~2–3 Ct in comparison to the MS2-EC Ct of the corresponding individual sample based on the approach used in individual sample analysis.

—, no signal.

#### MS2 phage as an extraction control

Human RNAse P (RP) is recommended as an extraction control (human specimen control) to ensure all the steps in nucleic acid extraction work properly. RP also serves as a specimen collection control (5), indicating whether the specimen was collected (e.g., NPS) properly or whether a repeat collection may be needed (when the result is negative for N1, N2, and RP). RP may not offer any insight into extraction performance across samples because Ct values for RP vary significantly between samples. The nucleic acid extraction step is a very critical part of the workflow in real-time PCR-based molecular detections, particularly in high-throughput environments, and must be closely monitored. A fixed amount of externally spiked control, such as RNA bacteriophage MS2, is effective (52–54) in monitoring the nucleic acid extraction and the subsequent reverse transcription step in the process. MS2 spike as an external control was also used in other SARS-CoV-2 surveillance approaches (45, 55). We included the MS2 spike as a control for monitoring the extraction performance in the SARS-CoV-2 test process.

#### TaqMan FAST virus 1-step MM is an effective alternative to TaqPath 1-step MM

**Figure 2:**
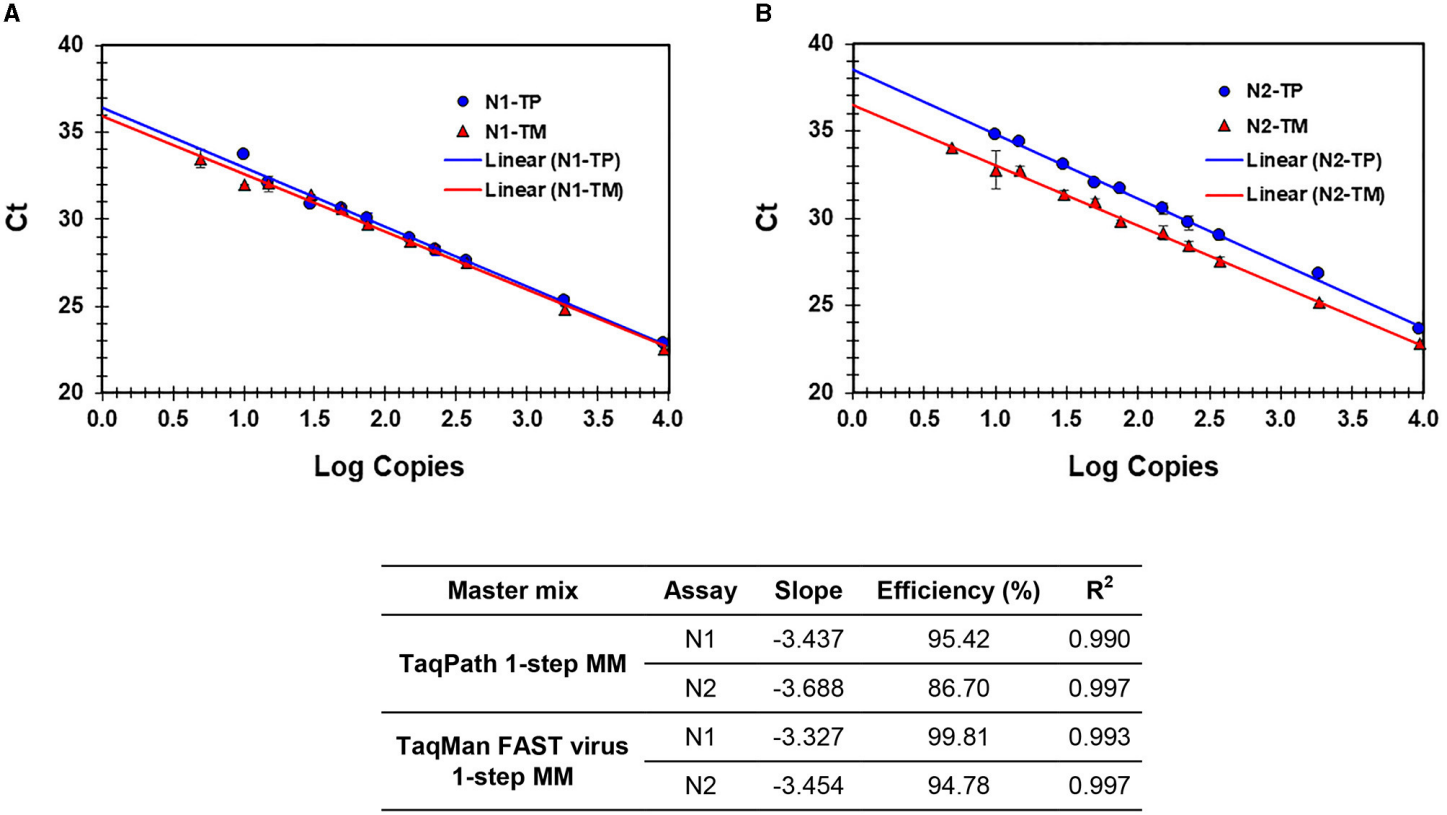
**(A, B)** Process efficiency of SARS CoV-2 detection in simulated pools prepared in VTM. Linear regression analysis of SARS-CoV-2-specific N1 and N2 Ct values plotted against the Log copies. Results for N1 **(A)** and N2 **(B)** are presented. N1-TP and N2-TP: Ct values for N1 and N2, respectively, in TaqPath 1-step real-time RT-qPCR MM. N1-TM and N2-TM: Ct values for N1 and N2, respectively, in TaqMan FAST virus 1-step MM. The table shows the slope, efficiency, and *R*^2^ values.

**Figure 3:**
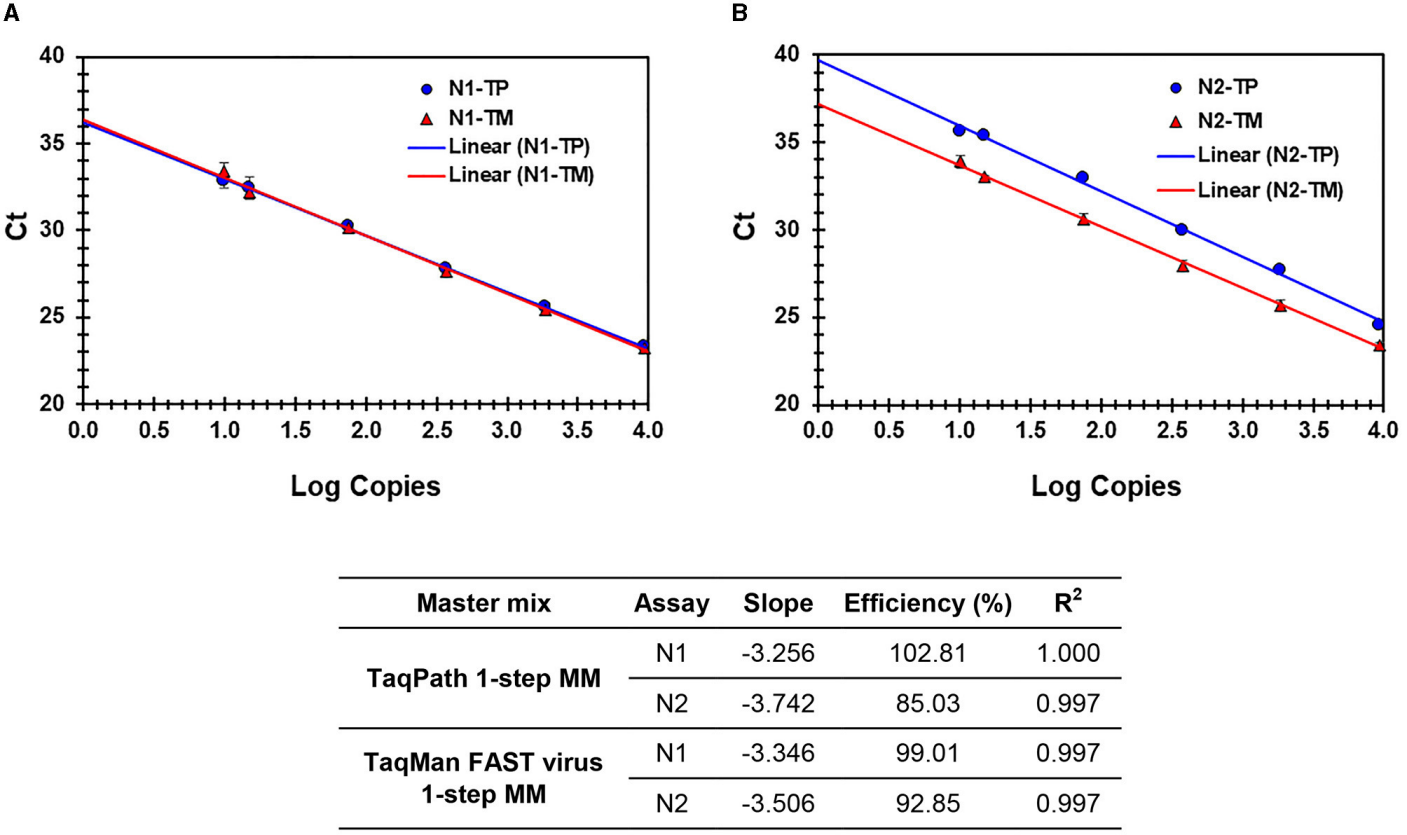
**(A, B)** Process efficiency of SARS CoV-2 detection in simulated pools prepared in saliva. Linear regression analysis of SARS-CoV-2-specific N1 and N2 Ct values plotted against the Log copies. Results for N1 **(A)** and N2 **(B)** are presented. N1-TP and N2-TP: Ct values for N1 and N2, respectively, in TaqPath 1-step real-time RT-qPCR MM. N1-TM and N2-TM: Ct values for N1 and N2, respectively, in TaqMan FAST virus 1-step MM. The table shows the slope, efficiency, and *R*^2^ values.

A performance comparison of TaqPath™ 1-Step RT-qPCR Master Mix (TP-MM) and TaqMan FAST Virus 1-step Master Mix (TM-MM) for testing SARS-CoV-2 N1 and N2 assays showed (Table 4) both master mixes worked well, while the N2 assay resulted in lower Ct values with TM-MM on all instruments tested. The observed Ct difference [Ct _(TP − MM)_ – Ct _(TM − MM)_] for N2 (mean ± standard deviation; SD) is 1.42 ± 0.2, 1.47 ± 0.33, and 0.74 ± 0.07 for ABI 7500 Dx and QuantStudio-6/96 well and 384 well, respectively. This difference becomes more obvious with results from standard curve experiments (Figures 2A, B, 3A, B), which show that N2 has better performance with TM-MM (see below).

**Table 4:** Performance of SARS-CoV-2-specific N1 and N2 assays in two different master mixes.

		**TaqPath**™ **1-step MM**		**TaqMan**™ **FAST Virus 1-step MM**	
**Instrument**	**Assay**	**Mean Ct**	**S.D**.	**Mean Ct**	**S.D**.
ABI 7500 Dx	**N1**	29.85	0.10	29.85	0.05
QS-6/96 well	**N1**	30.19	0.00	29.37	0.20
QS-6/384 well	**N1**	30.54	0.20	30.32	0.19
ABI 7500 Dx	**N2**	31.27	0.12	29.85	0.22
QS-6/96 well	**N2**	30.96	0.21	29.49	0.25
QS-6/384 well	**N2**	31.49	0.04	30.75	0.06

Heat-inactivated SARS-CoV-2 virus was spiked into VTM and processed for nucleic acid extraction and real-time PCR (150 copies/qPCR). The data presented is the mean Ct ± SD of five replicates. QS-6 is QuantStudio-6.

#### Process efficiency and analytical sensitivity (LOD)

**Figure 4:**
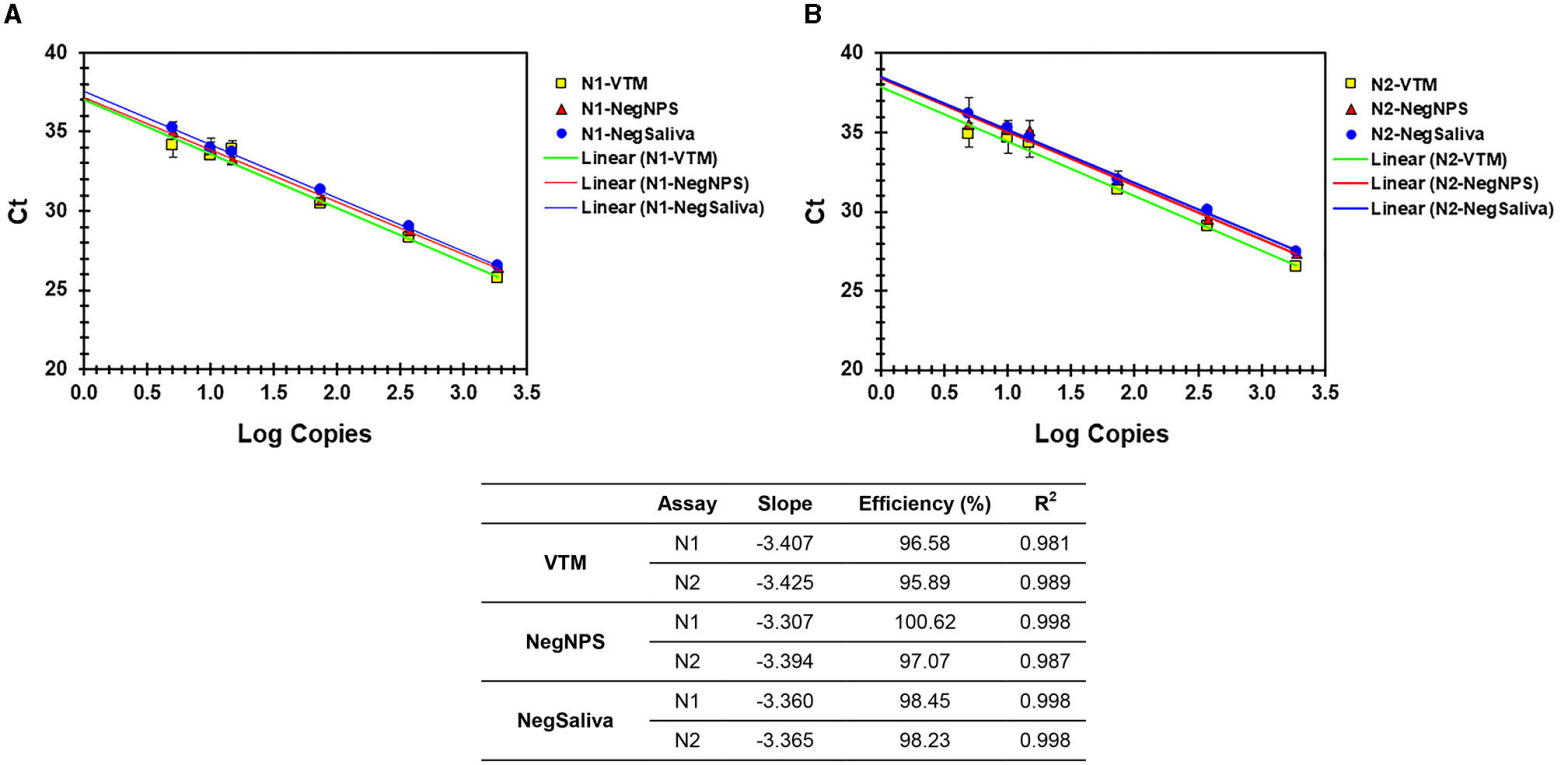
**(A, B)** Analytical efficiency of N1 and N2 assays with nucleic acids prepared from VTM, pooled negNPS, or pooled negsaliva as specimen matrix. Serial dilutions of heat-inactivated SARS-CoV-2 virus were prepared in different specimen matrices and processed for nucleic acid extraction followed by real-time PCR using TM-MM. Ct values were compared by linear regression analysis against the Log copies used. The table shows the slope, efficiency, and *R*^2^ values.

The sensitivity and linearity of this process were assessed by preparing serial dilutions (fivefold and twofold) of heat-inactivated virus in specimen medium (VTM/pooled Negative NPS/pooled negative saliva). For each dilution, a 40-μl aliquot (in triplicate) adjusted to 400 μl with specimen medium (simulated pool scenario) was processed for nucleic acid extraction and real-time PCR. Linear regression analysis of target (N1 and N2) specific Ct values and log copies showed that efficiencies are well within the acceptable range of ~95%−103% (for N1) and ~87%−98% (for N2) on different cyclers and master mixes tested. Standard curves generated with both master mixes reflect that the N1 assay showed no Ct difference between TP-MM and TM-MM, whether the virus was spiked into VTM or saliva (Figures 2A, 3A); however, the N2 assay with TM-MM resulted in lower Ct values (Figures 2B, 3B), suggesting that the N2 assay has better sensitivity with TM-MM. The observed Ct difference [Ct_(TP − MM)_ – Ct_(TM − MM)_] for N2 is in the range of ~0.89–2.02 in the VTM matrix and 1.14–2.34 in the saliva matrix, at the copies used to generate standard curves. This Ct difference tends to be more pronounced when template copies are low. In our experiments, we observed that the N1 assay has better sensitivity than the N2 assay, and this is also in agreement with reported observations (6, 56). Comparison of N1 and N2 standard curves also showed that whether the virus was spiked into pooled negative saliva, pooled negative NPS, or VTM (specimen collection medium), the entire process performed very similarly (Figures 4A, B), suggesting that the specimen matrix has no impact on the process efficiency.

The analytical sensitivity of this methodology was determined by LOD studies in two steps as recommended by the CDC protocol (5). Initially, an approximate LOD was estimated using dilutions of SARS-CoV-2 spiked into different specimen media (VTM, PBS, and pooled negative saliva). Based on the initial results, the final LOD of the process was determined by using 20 replicates each consisting of 40 μl of virus stock (80 copies) in a final volume of 400 μl (adjusted with appropriate specimen medium) and being processed for nucleic acid extraction followed by real-time PCR. This methodology resulted in a LOD of 10 copies/qPCR (95% confidence interval), which corresponds to 2,000 copies/ml of an individual specimen (Table 5). Additionally, LOD (10 copies/PCR) remained the same whether we used TP-MM or TM-MM for real-time PCR on multiple instruments (ABI 7500, ABI 7500 Dx, and QuantStudio-6). QuantStudio-6 with a 384-well block can offer high-throughput capability.

**Table 5:** Limits of detection (LOD) of pooling-based methodology.

				**TaqPath 1-step MM**			**TaqMan FAST Virus 1-step MM**			
**Matrix**	**Copies/ PCR**	**Copies/ mL**	**Target**	**# Pos./ total**	**Mean Ct** ± **S.D**.	**Median Ct**	**# Pos./ total**	**Mean Ct** ± **S.D**.	**Median Ct**	**Instrument**
VTM	10	2,000	N1	19/20	34.46 ± 0.99	34.38	20/20	34.11 ± 1.04	33.86	QS-6
			N2	19/20	37.06 ± 0.98	37.39	20/20	34.91 ± 1.02	34.86	
			N1	20/20	32.72 ± 0.58	32.65	20/20	32.58 ± 0.54	32.59	ABI-7500 Dx
			N2	20/20	35.72 ± 1.29	35.62	20/20	33.20 ± 0.68	33.10	
			N1	20/20	32.82 ± 0.92	32.60	20/20	32.84 ± 0.72	32.89	ABI-7500
			N2	20/20	34.86 ± 1.03	34.78	20/20	33.01 ± 0.58	32.96	
Saliva	10	2,000	N1	20/20	33.87 ± 0.61	33.80	20/20	34.15 ± 1.36	33.67	QS-6
			N2	19/20	36.57 ± 1.21	36.79	20/20	36.22 ± 0.72	36.10	
			N1	20/20	33.41 ± 0.82	33.42	20/20	34.31 ± 0.81	34.40	ABI-7500 Dx
			N2	20/20	35.57 ± 0.57	35.51	20/20	36.00 ± 0.92	35.88	
			N1	20/20	32.97 ± 0.59	32.94	20/20	34.36 ± 0.88	34.09	ABI-7500
			N2	20/20	36.59 ± 0.72	36.39	20/20	34.59 ± 0.66	34.60	
PBS	10	2,000	N1	20/20	34.07 ± 0.87	34.10	ND	ND	QS-6
			N2	19/20	36.10 ± 0.87	35.79	ND	ND	
			N1	20/20	34.00 ± 1.00	33.71	20/20	33.67 ± 0.79	33.50	ABI-7500 Dx
			N2	20/20	35.30 ± 0.67	35.17	20/20	34.20 ± 0.61	34.26	
			N1	ND	ND	ND	ND	ABI-7500
			N2	ND	ND	ND	ND	

The analytical sensitivity (LOD) of the pooling-based methodology was determined by preparing 20 replicate samples with each specimen medium and processed them for nucleic acid extraction and real-time PCR using TP-MM and TM-MM on three different real-time PCR cyclers.

VTM, viral transport medium; PBS, phosphate-buffered saline; QS-6, QuantStudio-6; ND, not done.

#### Saliva is as good a specimen as NPS for SARS-CoV-2 detection

**Figure 5:**
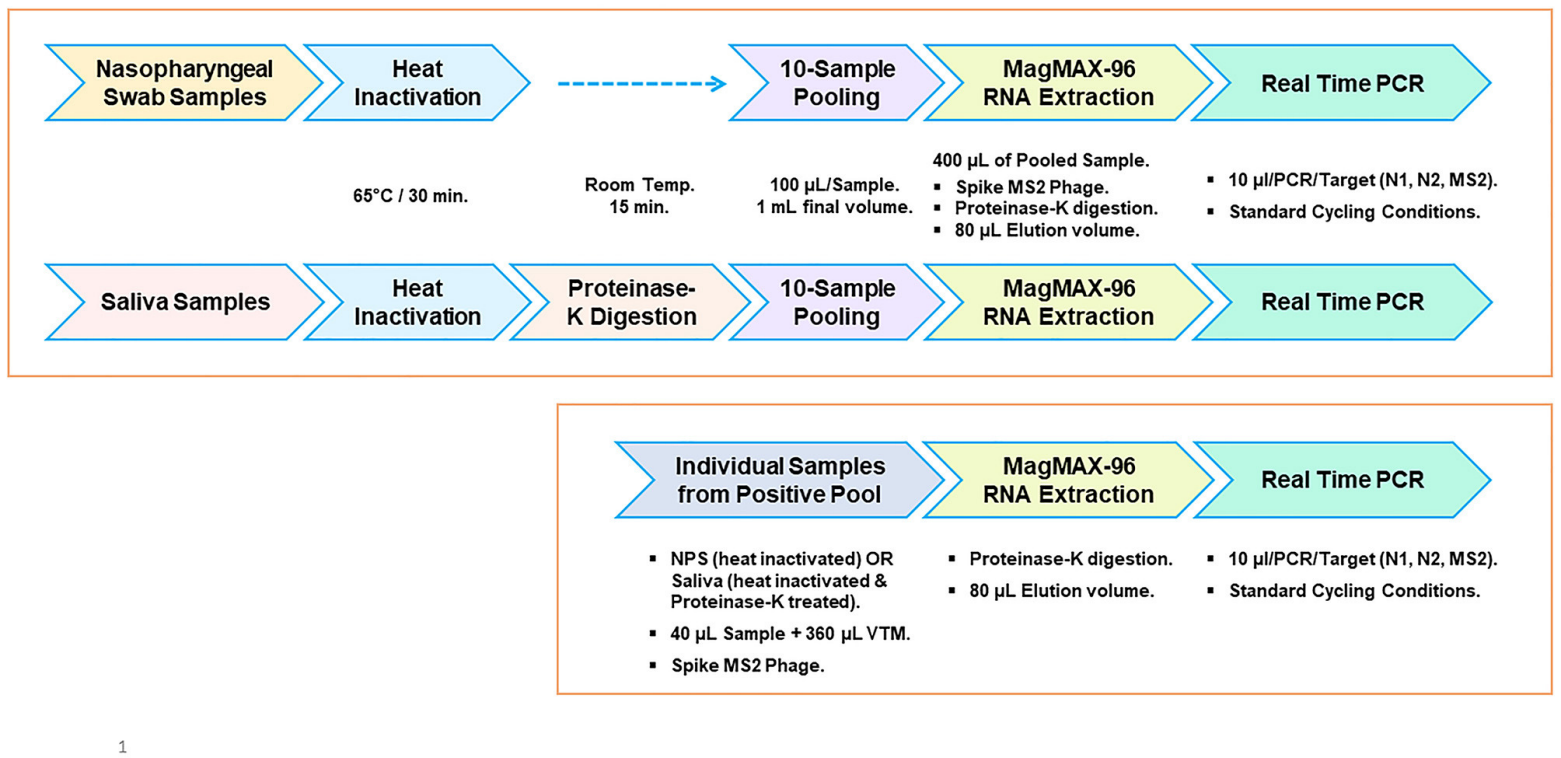
Workflow outline for surveillance screening for SARS-CoV-2. Pooled testing of NPS and saliva specimens **(top panel)**. Workflow for individual sample analysis from positive pools **(bottom panel)**.

We evaluated the feasibility of using saliva as the specimen medium for this pooling-based methodology. Saliva samples are intrinsically viscous, and accurate dispensing is a major issue in specimen pooling; hence, a proteinase K pretreatment step was included in the process to enable accurate pipetting for pooling. Serial dilutions of the virus spiked into pooled negative saliva, VTM, and pooled negative NPS performed similarly and resulted in highly comparable efficiencies (Figures 4A, B), maintaining the same analytical sensitivity (Table 5). The entire process from sample to result involves the same steps for NPS specimens and saliva specimens, except for an additional proteinase K treatment step prior to saliva pooling (Figure 5). These results suggest that this methodology is also well-suited for saliva specimens in addition to NPS samples.

#### PBS as the transport medium

Due to the supply shortages of collection tubes containing VTM early in the pandemic and to add flexibility to certain DA medical units, we evaluated PBS as an alternative transport medium. SARS-CoV-2 spiked into PBS showed comparable performance to the same amount of virus spiked into VTM without any significant difference in the Ct values or in the LOD achieved (Table 5; 10 copies/qPCR), suggesting the suitability of PBS as an alternate transport medium. We also evaluated the stability/detectability of the virus stored at +4°C in VTM or in PBS and found that the virus is stable in both media for at least 96 h upon storage at +4°C suggesting that samples can be stored at +4°C and pooled without any loss of sensitivity (Supplementary Table 1). Although we tested for up to 96 h only (at +4°C), a comprehensive study showed that the virus is stable for longer than 96 h in PBS (57).

#### NPS samples collected in PBS and VTM can be combined for pooled testing

While preparing the pools, it is likely that we may have some specimens in VTM and others in PBS. We verified the compatibility of preparing 10-sample pools from samples collected on these two different types of transport media. Different ratios of VTM and PBS (Ratio of % VTM vs. % PBS = 80:20; 60:40; 40:60; and 20:80) mixtures were spiked with known copies of the heat-inactivated SARS-CoV-2 virus. Nucleic acids were extracted and analyzed by real-time PCR. Our results suggest that pools prepared can have some samples collected in VTM and some in PBS (Supplementary Table 2).

## Discussion

Pooled testing is an effective way to expand analytical capacity in large-scale SARS-CoV-2 testing. A major drawback of most pooling methods (16–19, 25, 26) is the loss of sensitivity due to sample dilution and the potential for false negatives, particularly when the viral load in the sample is at or near the limit of LOD of the assay. Several studies addressed whether group testing is possible in clinical and public health settings to increase analytical capacity. Early studies using clinical samples addressed whether an individual positive sample would also be positive as part of a pool (16–19). Despite the observed Ct increase due to pooling (loss of sensitivity/dilution effect), their results showed specimen pooling maintained diagnostic sensitivity and suggested that pooled testing can be effective and increase the test capacity in low-prevalence situations. This is based on the positive agreement between the pool vs. individual test results and not so much on whether there is a Ct shift. Generally, clinical samples tend to have very high viral loads (low Ct values), and for samples with a high viral load (≥pool size × LOD), the sample dilution may not impact the final readout (positive pool vs. individual positive); however, when the viral loads are low and close to the LOD, the potential for false negatives due to sensitivity loss is a concern in pooled testing (16, 18, 21). Most pooling studies focus more on reporting the outcome of their surveillance analysis than developing a robust approach that any laboratory can adopt in a high-throughput situation. Our study addresses developing a methodology to mitigate the issue of sensitivity loss due to pooled testing. This methodology is developed such that any laboratory can apply this approach and avoid the possibility of false negatives, and it has a built-in ability to change the pool size (of up to 10 samples), offering additional versatility to researchers.

Detection methods developed for SARS-CoV-2 are intended for testing samples individually (un-pooled). These methods typically use a fixed sample input volume and elution volume during viral RNA extraction (Figure 1; Panel-I). Such a 1:1 relationship between the sample input volume and the nucleic acid elution volume is a key aspect of the well-established CDC 2019-nCoV Real-Time RT-PCR diagnostic panel (5). Adopting methods meant for single-sample analysis for pooled testing by mixing equal volumes of multiple samples leads to sample dilution (Figure 1; Panel-II, Method-A); however, if the initial dilution resulting from multi-sample pooling is compensated by appropriate concentration step(s), then such an approach would overcome the dilution effect. In this study, we employed this strategy through seamless concentration during the nucleic acid extraction and in real-time PCR (Figure 1; Panel-II, Method-B). We evaluated this by using controlled laboratory-created pools as well as surveillance samples and demonstrated that loss of sensitivity due to pooling can be avoided.

Our validation studies clearly demonstrated that this pooling approach is effective in mitigating the dilution effect and results in no loss of sensitivity (Tables 1–3). A single positive in a 10-sample pool resulted in a Ct value comparable to that of the individually analyzed positive sample. This approach can be used with a pool size of up to 10 samples and allows the selection of an appropriate pool size depending on the disease prevalence by adjusting the input and elution volumes appropriately without compromising sensitivity. For example, this could be done with five-sample pooling or with a binary approach, analyzing positive 10-sample pools further as two sub-pools of 5 each (58). Pools with size larger than 10 have not been evaluated in our study. Unless the disease prevalence is extremely low, pooling samples in such high numbers (>10) may not be beneficial (27–29). Successful implementation of group testing relies on key parameters such as the LOD of the process, the sensitivity of the methodology used, and knowledge of disease prevalence to select the appropriate pool size. The use of extracted RNA to assess the efficiency or LOD does not take the nucleic acid extraction step into consideration (6); hence, the efficiency may not reflect the entire process's performance. We evaluated the efficiency and analytical sensitivity of the entire process starting from the sample and achieved a LOD of 10 copies/qPCR (95% confidence interval, CI), which is comparable to the LOD (~15.8 copies/PCR) reported by the CDC (5). Process efficiencies for N1 and N2 assays are well within an acceptable range (59, 60). Our analysis also included multiple real-time PCR instruments and two different master mixes (TP-MM and TM-MM) to provide much-needed flexibility while maintaining the same analytical sensitivity and similar efficiency, demonstrating the robustness of the process and assay performance. The N1 assay performed equally well in both master mixes at all the concentrations of the virus tested, while the N2 performed better in TM-MM and is a preferred alternative to TP-MM (based on our studies), at least for surveillance purposes. Earlier studies also reported that TM-MM is suitable for SARS-CoV-2 testing by real-time PCR (61, 62). SARS-CoV-2 spiked into multiple specimen media (VTM, pooled negative NPS, and pooled negative saliva) resulted in the same analytical sensitivity (Table 5) and high concordance in standard curve results (Figure 4), suggesting that saliva is as good a specimen as NPS for detecting the virus by this approach. In addition to avoiding the loss of sensitivity, this comprehensive methodology (Figure 5) has several clear advantages: (a) it is well optimized using a reference virus of known copies; (b) it uses the CDC protocol (5) for single sample analysis as a benchmark; (c) it includes a heat inactivation as the first step (62–65) to ensure safer handling of specimens and minimize the risk to the personnel while preparing multi-sample pools; (d) it includes MS2-EC to closely monitor the nucleic acid extraction step of the process; and (e) it is compatible with automation.

Pooling multiple swabs into one tube is another method that can avoid the dilution effect (22–24); however, this approach requires the collection of two swabs from every individual initially or to retest individuals from a positive pool. Collecting two swabs is very inconvenient and uncomfortable for the individual. Analysis of pools with varying Ct values prepared by spiking SARS-CoV-2 positive specimens into a SARS-CoV-2 negative matrix (pool size = 10) showed that there is no loss of sensitivity (20, 21). In one study (21), the researchers observed a false negative rate of 13.3% when Ct values were >35. A rigorous evaluation of LOD or linearity (using standard curves) is difficult with clinical samples where the copy numbers of the virus are not known other than Ct values. Our methodology used a reference virus of known copies to develop comprehensive validation data to establish the proof of concept, LOD, and process efficiency with the same controlled reagents and workflow process for analyzing individual samples or pools. The CDC has provided recommendations and guidance to mitigate sensitivity loss in pooled testing for diagnostic use (5). It recommends using a pool size of no more than four samples and adjusting the input volume and elution volume of the 4-sample pool to compensate for the dilution effect. It also provides qualitative test results for individual vs. pooled specimens. In contrast to the CDC-recommended pooling method, which is intended for diagnostic use, the primary objective of our methodology is to introduce the capability to pool up to 10 samples to allow high-throughput screening of asymptomatic individuals for public health purposes.

An important concern with real-time PCR-based pooled testing involves samples, either as un-pooled individuals or as pools, with Ct values in the range of >LOD and < Ct 40. These Ct values fall outside of the LOD (95% CI) and will have a much lower detection CI; therefore, depending on the percentage of such data points, the overall outcome may be impacted. Over the course of a pandemic, the Ct value distribution changes considerably, and Ct values tend to be higher when the positivity rate goes down, indicating a lower viral load (66). Technical issues associated with assembling real-time PCR can also introduce variables, particularly for samples with a very low viral load (>LOD). Instead of testing each target (N1, N2, and RP or MS2) with a single RT-qPCR test/sample (pool or individual), a multiplex PCR with three replicates would increase the robustness of the test while maintaining the same number of tests per sample. This is one way to mitigate such technical variables. It is possible to integrate automation into our method by employing a liquid-handling robot for specimen pooling and for real-time PCR set-up. Our methodology clearly demonstrates that by appropriately concentrating the nucleic acid as a part of the process, one can avoid the dilution effect and loss of sensitivity. The underlying framework of this approach can also be applied in designing a methodology for pooling-based large-scale surveillance efforts in combating future pandemics.

Our study has some limitations. We used a limited number of surveillance samples in the form of NPS to demonstrate the proof of principle. We do not have a large collection of either NPS or saliva samples to conduct a retrospective study in a real-life situation to glean data on its performance in a high-throughput environment. While this is the intended purpose, at present we do not have any data on our method's real-life performance. Second, we have not evaluated our methodology on other nucleic acid extraction platforms except the MagMAX 96/KingFisher Flex systems. It is possible that some laboratories may not have this instrument; however, we believe that in principle, any magnetic bead-based nucleic acid extraction platform that can accept 0.5–1.0 ml of sample input with an elution volume of 50–100 μl capacity, such as the Roche MagNA Pure 96, can be used.

In the context of public health within the Department of the Army, through which this study was funded, screening all asymptomatic service members regularly in a clinical setting would require large amounts of diagnostic-grade reagents and would likely exceed available clinical testing capacity, as was often observed during the COVID-19 pandemic. This urgent testing need was met successfully by implementing our screening approach in 10-sample pools. This pool size allowed us to screen Soldiers in small groups for the presence of SARS-CoV-2, and only individuals from the positive pools were then required to undergo clinical testing. This method received accreditation from the American Association for Laboratory Accreditation (A2LA) and was implemented as a part of the comprehensive SARS-CoV-2 surveillance effort (67) at the Army Public Health Center (APHC), presently the Defense Centers for Public Health-Aberdeen (DCPH-A).

## Data availability statement

The original contributions presented in the study are included in the article/Supplementary material, further inquiries can be directed to the corresponding author.

## Ethics statement

The studies involving human participants were reviewed and approved by Office of the Human Protection. The patients/participants provided their written informed consent to participate in this study.

## Author contributions

GM and SP: carried out experiments and analysis. AG and WK: manuscript editing and suggestions. RT: conceptualization, support, manuscript editing, and suggestions. SY: conceptualization, experimental design, data analysis, interpretation, and manuscript preparation. All authors contributed to the article and approved the submitted version.

## Supplementary material

Supplementary Table 1 SARS-CoV-2 virus is stable in VTM or PBS for at least 96 h at +4°C. Known copies of SARS-CoV-2 spiked (80 copies/400 μl) into VTM or PBS and stored at +4°C for 24 and 96 h. Control tubes were not stored and processed immediately. At the end of 24 h and 96 h of storage, these samples were processed for nucleic acid extraction and real-time PCR using SARS-CoV-2-specific N1 and N2 assays. The experiment was performed with five replicates for each time point (80 copies/400 μl equals 10 copies/qPCR). The data presented here are mean ± SD. We used TaqPath 1-step MM and an ABI 7500 Dx real-time PCR cycler.

Supplementary Table 2 Pooling of NPS specimens collected both in VTM and PBS. While preparing the pools, it is likely that we will have some specimens in VTM and others in PBS. We verified whether 10-sample pools can be prepared by combining samples collected in these two different types of transport media. Different ratios of VTM and PBS (ratio of % VTM vs. % PBS = 80:20; 60:40; 40:60; 20:80) were prepared, and 400 μl of such a mixture was spiked with 80 copies of the heat-inactivated SARS-CoV-2 virus. The extracted nucleic acids were eluted into 80 μl of elution buffer and used 10 μl (equal to 10 copies, which is at LOD) each for N1- and N2-specific real-time PCR.
