# Supplementary material for: A novel strategy to avoid sensitivity loss in pooled testing for SARS-CoV-2 surveillance: validation using nasopharyngeal swab and saliva samples
Source: Front Public Health. 2023 Aug 10;11:1190308. doi: 10.3389/fpubh.2023.1190308 (PMC10450028; doi:10.3389/fpubh.2023.1190308)
Supplement: Supplementary Table 1 — SARS-CoV-2 virus is stable in VTM or PBS for at least 96 h at +4°C. Known copies of SARS-CoV-2 spiked (80 copies/400 μl) into VTM or PBS and stored at +4°C for 24 and 96 h. Control tubes were not stored and processed immediately. At the end of 24 h and 96 h of storage, these samples were processed for nucleic acid extraction and real-time PCR using SARS-CoV-2-specific N1 and N2 assays. The experiment was performed with five replicates for each time point (80 copies/400 μl equals 10 copies/qPCR). The data presented here are mean ± SD. We used TaqPath 1-step MM and an ABI 7500 Dx real-time PCR cycler. [file Table_1.docx]

**Supplementary Table-1**

|  |  | **Mean Ct ± S.D. (10 copies/PCR)** | |
| --- | --- | --- | --- |
|  |  | **VTM** | **PBS** |
| **N1** | **0-Hrs.** | 34.53 ± 0.56 | 33.82 ± 0.48 |
|  | **24-Hrs.** | 33.96 ± 0.46 | 34.07 ± 0.48 |
|  | **96-Hrs.** | 34.47 ± 0.78 | 34.01 ± 0.29 |
| **N2** | **0-Hrs.** | 35.55 ± 0.45 | 35.50 ± 0.27 |
|  | **24-Hrs.** | 35.10 ± 0.29 | 34.87 ± 0.52 |
|  | **96-Hrs.** | 35.80 ± 0.59 | 34.70 ± 0.44 |
