# Supplementary material for: A novel strategy to avoid sensitivity loss in pooled testing for SARS-CoV-2 surveillance: validation using nasopharyngeal swab and saliva samples
Source: Front Public Health. 2023 Aug 10;11:1190308. doi: 10.3389/fpubh.2023.1190308 (PMC10450028; doi:10.3389/fpubh.2023.1190308)
Supplement: Supplementary Table 2 — Pooling of NPS specimens collected both in VTM and PBS. While preparing the pools, it is likely that we will have some specimens in VTM and others in PBS. We verified whether 10-sample pools can be prepared by combining samples collected in these two different types of transport media. Different ratios of VTM and PBS (ratio of % VTM vs. % PBS = 80:20; 60:40; 40:60; 20:80) were prepared, and 400 μl of such a mixture was spiked with 80 copies of the heat-inactivated SARS-CoV-2 virus. The extracted nucleic acids were eluted into 80 μl of elution buffer and used 10 μl (equal to 10 copies, which is at LOD) each for N1- and N2-specific real-time PCR. [file Table_2.docx]

**Supplementary Table-2**

| **% V/V** | **100% VTM** | **80% VTM**  **20% PBS** | **60% VTM**  **40% PBS** | **40% VTM**  **60% PBS** | **20% VTM**  **80% PBS** | **100% PBS** |
| --- | --- | --- | --- | --- | --- | --- |
| **N1** | 34.28 ± 0.10 | 34.82 ± 0.47 | 34.22 ± 0.00 | 33.85 ± 0.60 | 35.27 ± 1.28 | 34.07 ± 0.13 |
| **N2** | 35.59 ± 0.04 | 35.15 ± 0.39 | 35.30 ± 0.53 | 35.55 ± 0.23 | 34.97 ± 0.01 | 36.09 ± 0.06 |
